# Hair Cell Generation in Cochlear Culture Models Mediated by Novel γ-Secretase Inhibitors

**DOI:** 10.3389/fcell.2021.710159

**Published:** 2021-08-13

**Authors:** Silvia T. Erni, John C. Gill, Carlotta Palaferri, Gabriella Fernandes, Michelle Buri, Katherine Lazarides, Denis Grandgirard, Albert S. B. Edge, Stephen L. Leib, Marta Roccio

**Affiliations:** ^1^Neuroinfection Laboratory, Institute for Infectious Diseases, University of Bern, Bern, Switzerland; ^2^Cluster for Regenerative Neuroscience, Department for BioMedical Research (DBMR), University of Bern, Bern, Switzerland; ^3^Laboratory of Inner Ear Research, Department for BioMedical Research (DBMR), University of Bern, Bern, Switzerland; ^4^Graduate School for Cellular and Biomedical Sciences, University of Bern, Bern, Switzerland; ^5^Audion Therapeutics B.V., Amsterdam, Netherlands; ^6^Massachusetts Eye and Ear, Boston, MA, United States; ^7^Harvard Medical School, Boston, MA, United States; ^8^Harvard Stem Cell Institute, Cambridge, MA, United States; ^9^Department of Otorhinolaryngology, Head and Neck Surgery, University Hospital Zurich, Zurich, Switzerland; ^10^Department of Otorhinolaryngology, University of Zurich, Zurich, Switzerland

**Keywords:** drug therapy, sensorineural hearing loss, cochlear organoids, hair cell regeneration, Notch signaling, gamma secretase inhibitor

## Abstract

Sensorineural hearing loss is prevalent within society affecting the quality of life of 460 million worldwide. In the majority of cases, this is due to insult or degeneration of mechanosensory hair cells in the cochlea. In adult mammals, hair cell loss is irreversible as sensory cells are not replaced spontaneously. Genetic inhibition of Notch signaling had been shown to induce hair cell formation by transdifferentiation of supporting cells in young postnatal rodents and provided an impetus for targeting Notch pathway with small molecule inhibitors for hearing restoration. Here, the oto-regenerative potential of different γ-secretase inhibitors (GSIs) was evaluated in complementary assay models, including cell lines, organotypic cultures of the organ of Corti and cochlear organoids to characterize two novel GSIs (CPD3 and CPD8). GSI-treatment induced hair cell gene expression in all these models and was effective in increasing hair cell numbers, in particular outer hair cells, both in baseline conditions and in response to ototoxic damage. Hair cells were generated from transdifferentiation of supporting cells. Similar findings were obtained in cochlear organoid cultures, used for the first time to probe regeneration following sisomicin-induced damage. Finally, effective absorption of a novel GSI through the round window membrane and hair cell induction was attained in a whole cochlea culture model and *in vivo* pharmacokinetic comparisons of transtympanic delivery of GSIs and different vehicle formulations were successfully conducted in guinea pigs. This preclinical evaluation of targeting Notch signaling with novel GSIs illustrates methods of characterization for hearing restoration molecules, enabling translation to more complex animal studies and clinical research.

## Introduction

Hair cells are the sensory receptors of the vestibular and the auditory system in the inner ear. In the cochlea there are two types of auditory hair cells: inner hair cells (IHCs), which are the primary sound detectors and outer hair cells (OHCs), which act as acoustic pre-amplifiers (Fettiplace and Hackney, 2006). Hair cells are organized in rows, intercalated by different types of supporting cells, which exert supporting, trophic and barrier functions. Degeneration of hair cells is the most common reason for sensorineural hearing loss and can be caused by acoustic overstimulation, ototoxic drugs, aging, genetic disorders or infections (Muller and Barr-Gillespie, 2015; Bommakanti et al., 2019). Disabling hearing loss affects the quality of life of 5% of the world population, with high social and economic costs (Wilson et al., 2017).

Non-mammalian vertebrates regenerate damaged or lost vestibular as well as auditory hair cells throughout life and thereby restore sensory functions (Corwin, 1981, 1985; Corwin and Cotanche, 1988; Ryals and Rubel, 1988; Weisleder and Rubel, 1993). This is mediated by the activity of supporting cells in the sensory patches, which give rise to new hair cells either through cell division or transdifferentiation. In mammals, some degree of spontaneous hair cell regeneration has been reported for the vestibular organs (Forge et al., 1993; Warchol et al., 1993; Sayyid et al., 2019; Wang et al., 2019). For the cochlear auditory epithelium, similar responses have been detected only in early postnatal stages, prior to hearing onset (Atkinson et al., 2015). Specifically, spontaneous regeneration has been observed in mice when hair cells were genetically ablated in a perinatal window of 4 days (Cox et al., 2014). Fate-mapping showed that the novel hair cells were generated from supporting cells by mitotic regeneration and direct transdifferentiation (Bramhall et al., 2014; Cox et al., 2014; Hu et al., 2016). Despite the limited extent of spontaneous regeneration, these findings have directed attention toward chemical and genetic modifications that might promote and extend the permissive window for regeneration.

The Notch and the Wnt signaling pathways have been recently targeted to enhance this regenerative potential (Roccio et al., 2019; Samarajeewa et al., 2019). A subset of supporting cells within the postnatal cochlear epithelium expresses the Wnt co-receptor and target Lgr5 (Chai et al., 2011; Chai et al., 2012; Shi et al., 2012; Zak et al., 2016), a marker of stem cells in several epithelial organs (Barker et al., 2013). Lgr5+ cells display enhanced Wnt responsiveness and forced activation of Wnt in these cells, through genetic modification or chemical stimulation, causes their proliferation (Jacques et al., 2012; Roccio et al., 2015; Samarajeewa et al., 2018) as well as their differentiation into hair cells (Bramhall et al., 2014; Shi et al., 2014; Hu et al., 2016). *LGR5* expression and Wnt responsiveness have also been confirmed in human fetal cochleas and cochlear organoid cultures (Roccio et al., 2018).

The Notch signaling pathway controls hair cell and supporting cell specification and plays a role in tissue regeneration. During cochlear development, hair cells and supporting cells arise from a common precursor within the cochlear prosensory domain (Kelley, 2006; Groves and Fekete, 2012). Notch ligand-receptor interaction between prosensory progenitors defines the mosaic organization of hair cells and supporting cells (Kelley, 2006; Kiernan, 2013). Binding of Notch ligands triggers the cleavage of the Notch receptor, allowing the translocation of the intracellular domain to the nucleus, which transcriptionally regulates developmental gene programs and inner ear cell patterning (Kopan and Ilagan, 2009; Bray, 2016). Notch activation leads to the repression of Atoh1, a key transcription factor controlling hair cell differentiation (Bermingham et al., 1999; Chen et al., 2002; Woods et al., 2004), and otic progenitor cell fate is directed to supporting cell lineage in the absence of Atoh1 (Zheng et al., 2000; Zine et al., 2001; Hayashi et al., 2008; Doetzlhofer et al., 2009). Chemical or genetic inactivation of Notch signaling can induce hair cell formation by conversion of supporting cells (Korrapati et al., 2013; Mizutari et al., 2013; Li et al., 2015; Maass et al., 2015; Ni et al., 2016b). This has been made evident also in human prosensory cell-derived organoid cultures as chemical inhibition of Notch signaling enhanced hair cell differentiation, supporting the presence of similar mechanisms in human inner ear specification (Roccio et al., 2018).

Notch receptors are cleaved in the intracellular domain by enzymes known as γ-secretases (Kopan and Ilagan, 2009; Andersson and Lendahl, 2014; Bray, 2016). Pharmacological γ-secretase inhibitors (GSIs) have been tested in postnatal animal models as a strategy to induce hair cell differentiation and hearing restoration *in vivo* (Mizutari et al., 2013; Tona et al., 2014; Maass et al., 2015). In adult animals deafened by noise overexposure, inhibition of Notch signaling by local delivery of a single dose of the GSI LY411575 resulted in new hair cell formation and small improvements in the cochlear function in the low frequency regions that were correlated with increased expression of Atoh1 (Mizutari et al., 2013).

The aim of this study was to evaluate and characterize two novel GSI compounds, hereafter CPD3 and CPD8, and their efficacy to modify hair cell gene expression and direct *de novo* hair cell differentiation. The results reported are based on a series of *in vitro* models, including Notch-dependent cell lines, explants of the organ of Corti (OC), novel whole cochlea cultures and cochlear organoids derived from the mouse and rat inner ear. GSIs were also evaluated in models of ototoxic damage, in order to probe the regenerative potential after hair cell loss. Finally, we evaluated different GSI-containing formulations for their permeability through the round window membrane and performed pharmacokinetic studies to quantify local concentrations in the perilymph. These preclinical data supported the advancement of CPD3 to clinical trial. The assays presented in this study illustrate furthermore a potential pipeline for *ex vivo* characterization of hearing restoration molecules.

## Materials and Methods

### Animals

Animal studies (*in vivo* or *ex vivo*) were all approved by either the Animal Care and Experimentation Committee of the Canton of Bern, Switzerland [license BE 142/16 (SLL); BE119/12 (MR)] and followed the Swiss national guidelines for the performance of animal experiments, or performed under an approved institutional protocol according to National Institutes of Health guidelines, or under animal protocols approved by the Animal Studies Committee of Languedoc Roussillon that comply with French legislation and European Directives.

Female albino Hartley guinea pigs, 250 g, from Charles River (France), housed in macrolon cages (2/cage; Innocage Rat Static-Innovive, Paris) with filter air, were maintained at 22 ± 2°C on a 12-h light cycle with standard A04C (UAR) diet and water “*ad libitum*.” Lgr5-GFP mice containing an EGFP-IRES-CreERT2 knock-in allele at the Lgr5 locus were obtained from the Jackson Labs (Stock 008875, C57BL/6J/RccHsd background). Atoh1-nGFP transgenic mice (C57Bl/6J background) were from Jane Johnson (Lumpkin et al., 2003). Wistar rats were obtained from Charles River.

### Pharmacokinetics in the Inner Ear

γ-secretase inhibitors pharmacokinetic studies tested CPD3, CPD8 (Eli Lilly) or LY411575 (Stemcell Technologies) each formulated at 3.4 mg/ml in the following vehicles, 10% Poloxamer 338 (Sigma), 20% Poloxamer 338, 70% PEG400 (Sigma) or 1% hyaluronic acid (HA-700; Lifecore, Chaska, MN, United States) all prepared with PBS (pH 6.2). A single 70 μl injection of the formulated compound (total dose, 870 μg/kg) was delivered to the middle ear space of isoflurane-anesthetized guinea pigs by transtympanic administration. Perilymph from treated animals (perilymph volumes, >2 μl) was collected at time points up to 48 h post-administration (CILcare, Montpellier, France). Briefly, concentrations of each compound in perilymph and plasma, as well as controls prepared in artificial perilymph, were measured by LC-MS/MS on an AB Sciex, API 4000 mass spectrometer using an Onyx C18 5 μm 50^∗^2.0 mm separation column (Oroxcell, Romainville, France).

### Cell Lines and Tissue Culture

The human colon cancer cell line LS174T (CL-188; ATCC) was cultured without antibiotics in 1% FBS/Eagle’s Minimum Essential Medium (MEM; Thermo Fisher) (Asakura et al., 2018). The cells were exposed to GSIs or vehicle for 4 days, prior to analysis.

### Otic Sphere Assay

The generation of otic spheres followed previously described protocols (Oshima et al., 2009) with modifications during differentiation phase to test GSIs. Briefly, OCs were dissociated and single cells were deposited in ultralow-cluster plates (Corning) and expanded in “full otic medium” consisting of DMEM/F12, supplemented with N2, B27 (Thermo Fisher), EGF (20 ng/ml; Chemicon), bFGF (10 ng/ml; Chemicon), IGF-1 (50 ng/ml; Chemicon), and heparan sulfate (50 ng/ml; Sigma-Aldrich). After culturing for 3–4 days at 37°C, spheres were collected, plated in cell culture-treated 96 well-plate and cultured in DMEM/F12, supplemented with N2, B27 with/without GSIs for 7 days after which they were lysed for RNA extraction.

### Dissection and Isolation of Organ of Corti for Sensory Epithelium Explant Culture

OC explants were isolated from postnatal day 2 (P2) Wistar rats, P0-P2 mice (Lgr5-GFP or Atoh1-nGFP mice). Dissection of the cochlea under a stereomicroscope (Nikon SMZ800, Japan) allowed OC separation from stria vascularis and modiolus, and the sensory epithelium was plated with hair cells facing up on Cell-Tak (Corning, United States)-coated transwell-clear inserts (6-well format, 0.4 μm pore, Corning, United States). Dissection medium was exchanged for 1.5 ml full otic medium supplemented with 10% FBS (Invitrogen) and 0.01% Ampicillin (Sigma), added under the insert membrane, maintaining the OC under a thin film of medium during culture at 37°C. 5-ethyl-2′-deoxyuridine (EdU, 5 μM, Life Technologies) was added directly in the medium when indicated.

#### GSI-Treatment of Organotypic Cultures

Concentrations of small molecule GSIs {LY411575, CPD3, CPD8, DAPT (N-[N-(3,5-Difluorophenacetyl)-L-alanyl]-*S*-phenylglycine t-butyl ester; Sigma)} were prepared in DMSO and are indicated in Figure Legends and Results. DMSO included as Control. Treatment groups were assigned randomly.

#### Ototoxic Insults in Organotypic Cultures

To induce ototoxic damage, medium was removed from the OC cultures on the day after dissection and either 200 μM sisomicin (Sigma) in full otic medium, or 10^8^ cfu/ml *Streptococcus pneumoniae* (described below) in ampicillin-free and FBS-free otic medium were added under the transwell membrane (lower compartment) of the cultivation system. After 2 h of exposure to the ototoxic insult, OCs were carefully washed 3 times and culture was continued for 4 days in fresh full otic medium.

#### *Streptococcus pneumoniae* Inoculum

A clinical isolate of *S. pneumoniae*, serotype 3 was used as previously described (Leib et al., 2000; Perny et al., 2017). Briefly, bacteria were cultured overnight (ON) in brain-heart infusion medium and diluted 1:10 in fresh medium the following day. Bacteria were grown until the logarithmic phase was reached (after approximately 5 h). The culture was centrifuged for 10 min at 3,100 *g* at 4°C and the pellet was resuspended in 0.85% NaCl and centrifuged again. Bacteria were diluted to the desired optical density (OD_570 *nm*_) and the final inoculum concentration was determined by plating serial dilutions on Columbia sheep blood agar.

### *Ex vivo* Whole Cochlea Culture

To culture the intact cochlea of P0–P1 mice, the entire otic capsule (cochlea and semicircular canals) was carefully dissected. Connective tissues and nerves were removed to clear the round window niche and the capsule was positioned with the round window facing up in a sterile 35 mm dish. A surgical sponge was employed to blot dry the interior of the round window niche, which was then filled with 2 μl of the formulated drug and applied with a small pipette tip. Following 2 h of drug treatment at 37°C with 5% CO2, the tissue was transferred to a 50 ml conical tube containing 10 ml medium (DMEM, 5%FBS, 5% horse serum and ampicillin) and the tube was sparged with 95% O_2_/5% CO_2_ for 5–10 min and incubated for 2 days at 37°C, rotating on a roller/rocker (SCILOGEX MX-T6-S Analog Tube Roller) at 15–17 rpm.

### Dissection and Isolation of Organ of Corti Cells for Organoid Culture

On day 0, the cochlear sensory epithelium was dissected and the OC was separated from the modiolus and stria vascularis, as described above, from either Wistar rat pups (P2), Lgr5-GFP mice (P0–P2) or Atoh1-GFP mice (P0–P2). To separate the sensory epithelial layer from underlying mesenchyme and neurons, the OC was placed in a 100 μl drop of Matrisperse Cell Recovery Solution (Corning) for 1 h at RT, then delaminated with the aid of forceps. Gentle enzymatic dissociation of hair cells and supporting cells followed with 100 μl drop of TrypLE (Gibco), 20 min at 37°C, then mechanical trituration in DMEM/F12. The cell suspension was centrifuged for 5 min at 500 *g* at 4°C and resuspended in DMEM/F12 medium supplemented with B27 and N2 (Invitrogen), EGF (50 ng/ml), bFGF (50 ng/ml), IGF (25 ng/ml), CHIR99021 (GSK-3β inhibitor, 3 μM, Merck), valproic acid (HDAC inhibitor, 1 mM, Sigma), 2-phospho-L-ascorbic acid (280 μM, Stemcell Technologies) and 0.01% ampicillin (Sigma). Cells were plated at 2,000 cells per well on 96 wells plate (Corning Costar; ultra-low attachment, round-bottom) to allow aggregation at 37°C. On day 1, Matrigel (GF-depleted, Corning; 2% stock) was added to a final concentration of 0.18 mg/ml. On day 3, cells were transferred to 24 wells plate (ultra-low attachment; Corning Costar), pooling the organoid aggregates from 4 to 5 wells (96 well-plate) to one 24-well, culturing at 37°C and exchanging half medium and Matrigel every other day, On day 10, organoids were differentiated by replacing medium with DMEM/F12 supplemented with B27, N2, CHIR99021 (3 μM), and Notch inhibitors CPD3 (5 μM) or LY411575 (5 μM), and culture was continued as above for another 10 days.

#### Organoid Ototoxic Treatment

On day 20, differentiated organoids were exposed to sisomicin for 2 h at the concentration of 500 μM. The medium was then washed and replaced with differentiation medium with/without GSI for an additional 4 or 7 days. For propidium iodide (PI) uptake, on day 24 of *in vitro* differentiation, cells were incubated in the presence of PI (5 μg/ml) for 5 min and subsequently washed and fixed. Immunostaining was then performed as indicated below.

### Gene Expression by RT-PCR

Cells or tissue were lysed using RLT Lysis Buffer (Qiagen) and high purity RNA was prepared on the MagNA Pure instrument using MagNA Pure 96 Cellular RNA LV Kit (Roche). Reagents from Thermo Fisher were used for cDNA synthesis by reverse transcription using random hexamers. Resulting cDNA was RNAse H treated and amplified by TaqMan GEX mastermix and PCR primers and probes for human ATOH1 (Hs00944192_s1) and TBP (Hs00427620_m1), mouse Tbp (Mm00446971_m1), Atoh1 (Mm00476035_s1), Hes5 (Mm00439311_g1), Pouf4s (Mm04213795_s1), and Myo7a (Mm01274015_m1) on the Applied Biosystems QuantStudio 7 Flex Real-Time PCR System. Normalized to Tbp, relative gene expression fold-change was quantified and compared between experimental and control conditions by ΔΔ*C*t calculation.

### Immunohistochemistry and Imaging of Organ of Corti Explants

Following culture, explants were washed (PBS) and fixed for 10 min at room temperature (RT) with 4% paraformaldehyde (PFA). Samples were permeabilized with 3% Triton X-100 for 30 min at RT and blocked with blocking buffer containing 2% bovine serum albumin (BSA) and 0.01% Triton X-100 for 1 h at RT.

Dividing cells were labeled with, 5-ethyl-2′-deoxyuridine (EdU) and detected with Click-it Plus kit (Life Technologies). Primary antibodies [mouse anti-Pou4f3 (1:200; Santa Cruz Biotechnology, United States) and rabbit anti-Sox2 (1:200; Cell Signalling Tech, United States)] were incubated in blocking buffer ON at 4°C. Samples were washed and incubated for 2 h at RT with secondary antibodies [goat anti-mouse Alexa Fluor 488 and goat anti-rabbit Alexa Fluor 555 (1:500 dilution, Invitrogen, United States)], as well as Phalloidin-Alexa Fluor 647 (Sigma). Explants were washed and mounted on glass slides with Fluoroshield containing DAPI (Sigma).

Explants were visualized with a Nikon Eclipse Ti-E for overview purposes. Individual *z*-planes at an interval of 5 μm were acquired with a confocal laser-scanning microscope (Zeiss LSM710, Plan-Apochromat 20×/0.8 NA objective) to ensure visualization of all hair cells.

Image processing was performed with the open-source image processing software FIJI, version 2.0 (Schindelin et al., 2012). Hair cells were quantified by counting Pou4f3 or Myo7a positive cells of the organ of Corti at three random microscopic fields for each cochlear region (base, middle, and apex) covering in total at least 1,200 μm. The number of hair cells (IHCs and OHCs) is expressed as unit per 100 μm.

### Immunofluorescent Staining and Imaging of Cochlear Organoids

For staining and imaging, organoids were allowed to adhere ON onto transwell-permeable support membranes (Corning) in 12-well plates coated with 100% Matrigel. The next day, they were fixed with 4% PFA (10 min). Cells were permeabilized with 3% Triton-X 100 for 10 min and incubated ON with blocking buffer containing 2% BSA and 0.01% Triton-X 100. Primary antibodies were incubated ON at 4°C (1:200 dilution). Anti-Sox2 (mouse, BD Bioscience), anti-Pou4f3/Brn-3c (mouse, Santa Cruz Biotechnology) anti-Myo7a (rabbit, Enzo Life Sciences) and anti-p27/Kip1 (rabbit, Abcam). Organoids were then rinsed and incubated ON with the secondary antibodies as described above. For FM1-43 uptake, organoids were first incubated with Hoechst (10 μg/ml), 30 min at 37°C, then a brief incubation FM1-43 (5 μM; Life Technologies) for 30 s, and washed twice before fixation (4% PFA). To image organoids, inserts were placed face down in a 24 well glass bottom plate (MatTek) and imaged on a Zeiss LSM710 laser scanning microscope (5 μm *Z*-stacks intervals). Compiled *Z*-projections were processed (FIJI, ver2.0) and hair cells quantified on individual planes with manual cell counter plugin, by evaluation of cytosolic expression and nuclear exclusion of Myo7a. Organoid volumes were estimated from the sum of the product of the area of the organoid (at each plane) and the *Z* stack height.

### Immunofluorescent Staining of Inner Ear Whole Mounts

For whole-mount immunohistochemistry, whole cochleae were fixed (4% PFA) ON at 4°C, then incubated in primary antibodies (72 h, rocking at 4°C), goat anti-GFP polyclonal Ab (AbCam, ab6673) and rabbit anti-Sox2 polyclonal IgG (GenTex, GTX101506) in 4% normal donkey serum. Following extended washes (over 24 h), cochlea were incubated for 24 h in secondary antibodies donkey anti-goat Alexa 488 (Thermo Fisher, A11055) and donkey anti-rabbit Alexa 647 (Thermo Fisher, A31573) and extensively washed. The intact OC were then microdissected from cochleae and mounted on a coverslip for confocal imaging. A LSM 510 Confocal Microscope (Zeiss) utilizing 63X Zeiss Plan-APOCHROMAT DIC oil, 1.4NA and Zen 2009 LSM imaging software was used to optically scan mid-apex regions of OCs. Cellular quantification and image analysis computation were performed with Imaris 8.2.1 software (Bitplane).

### Data Analysis and Statistics

Statistical analysis was performed with GraphPad Prism software (Prism9; GraphPad Software Inc., San Diego, CA, United States). For explant cultures, when the number of explants/replicates was not sufficient to test for Gaussian distribution normal distribution of the data was assumed based on previous experiments (Perny et al., 2017; Erni et al., 2019). Alternatively, the distribution of the datasets was tested by D’Agostino and Pearson normality test. Unpaired *t*-tests were used to compare parametric data sets of two groups. Welch’s correction was applied if variances were not equal. For non-parametric data, the Mann–Whitney test was adopted. One-way ANOVA was used to determine the difference in means among multiple groups and multiple comparison correction was tested. Graphs indicate the single data point, the mean values ± standard deviation. IC50s were calculated by non-linear regression of data normalized by percent change from controls to compare multiple experiments. Differences in IC50s from Best fit data were compared by Least Squares (*F*-test; Extra sum of squares; alpha = 0.05). The number of technical and biological replicates are indicated in Figure Legends.

## Results

### Activity and Inner Ear Pharmacokinetics of GSIs Examined in This Study

**FIGURE 1:**
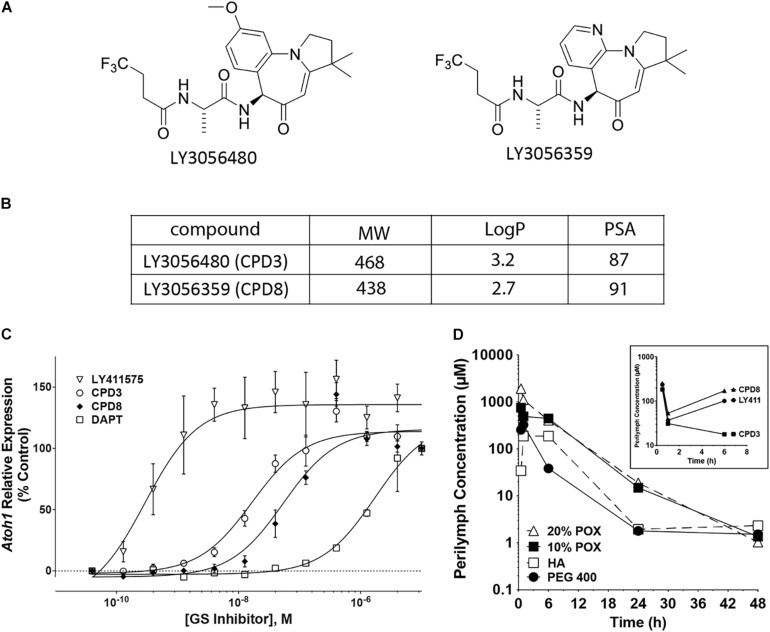
γ-secretase inhibitors (GSI) characterization. **(A)** Structures of the two novel GSIs tested in these studies. **(B)** Properties of the two GSIs (CPD3 and CPD8). Molecular weights (MW), logarithms of the octanol-water partition coefficients (logP), and polar surface areas (PSA). **(C)** Dose-response curves for CPD3, CPD8, DAPT, and LY411575 in the human intestinal epithelial cell line LS174T. Fold-change of human *ATOH1* expression in response to GSI treatment after 72 h. IC50s for the 4 GSIs were 17.3 nM (CPD3; *open circles*), 62.0 nM (CPD8; *filled diamonds*), 0.4 nM (LY411575; *open triangles*), and 1.4 μM (DAPT; open squares). Atoh1 expression is reported as fold-change relative to carrier control [0.05% dimethyl sulfoxide (DMSO)]. Duplicate measurements are from 4 separate experiments (total *n* = 8). **(D)** Inner ear pharmacokinetic study in albino Hartley guinea pigs. A single transtympanic dose of CPD3 in [20% poloxamer (POX; *open triangle*), 10% POX; *closed square*], hyaluronic acid (HA; *open square*), or PEG 400 (*closed circle*) resulted in detectable CPD3 concentrations in the perilymph up to 48 h post-dosing. A single transtympanic dose of CPD3 (*squares*), CPD8 (*triangles*), and LY411575 (*circles*) in 10% POX resulted in perilymph levels shown at 0.5, 1, and 6 h post-dosing (*inset graph*).

To measure the relative potency between different GSIs, a pharmacological assay was initially used to identify the dose ranges effective to induce *ATOH1* expression. The cell line LS174T, a human colorectal adenocarcinoma, was used to evaluate Notch-dependent expression of *ATOH1* (Kazanjian et al., 2010). Two compounds from Eli Lilly, designated LY3056480 (CPD3) and LY3056359 (CPD8) (Figures 1A,B) were compared with the commercially available GSIs LY411575 and DAPT. *ATOH1* expression was upregulated by GSI treatment in a dose-dependent manner after 72 h. IC50s for the 4 GSIs were 17.3 nM (CPD3; *open circles*), 62.0 nM (CPD8; *filled diamonds*), 0.4 nM (LY411575; *open triangles*) and 1.4 μM (DAPT; *open squares*). These IC50s ranked the potency for CPD3 and CPD8 intermediate between that of LY411575 and DAPT, with the CPD3 IC50 measured as significantly lower than that of CPD8 (Figure 1C; *p* < 0.05). The intermediate potency of CPD3 and 8 was likely to be adequate to achieve levels above the IC50 after dilution into perilymph in the inner ear.

To verify this, *in vivo* compound concentrations were measured in perilymph of guinea pigs after intratympanic delivery of GSIs into the middle ear. Initially, four vehicle formulations, with different physical properties, were tested, poloxamer 338 (POX; at 10% or 20%), hyaluronic acid (HA), or polyethylene glycol 400 (PEG). For this comparison only one GSI was prepared in each formulation (CPD3, 3.4 mg/ml) and a single dose was injected into the middle ear. Perilymph and plasma were sampled for 48 h (Figure 1D). The highest perilymph concentrations were achieved in 10% POX and 20% POX formulations, respectively. The mean, maximal perilymph concentration for these formulations was reached at 0.5 h post-dosing and declined over time. Detectable CPD3 remained in perilymph throughout the test, and at 48 h concentrations between 485 ng/ml (1.0 μM) and 1,000 ng/ml (2.1 μM) indicated sufficient residence times and concentrations for GSI activity. Next, using only 10% POX as vehicle, inner ear adsorption was compared between CPD3, CPD8, and LY411575 by transtympanic injections (Figure 1D, *inset*). Perilymph concentrations were sampled at 0.5, 1, and 6 h post-dosing, with concentrations of CPD3, CPD8, and LY411575 quantifiable at all time points. Each compound achieved levels several folds above their respective IC50s. The mean maximal perilymph concentration of CPD3 at 0.5 h post-dosing was 85,489 ng/ml (183 μM), then declined at 1 h to 12,928 ng/ml (27.7 μM), and at 6 h to 12,652 ng/ml (27.1 μM). GSIs in plasma samples were not detected (data not shown).

### GSI-Mediated Induction of Hair Cell Gene Expression and *de novo* Generation of Hair Cells in the Neonatal Cochlear Sensory Epithelium

**FIGURE 2:**
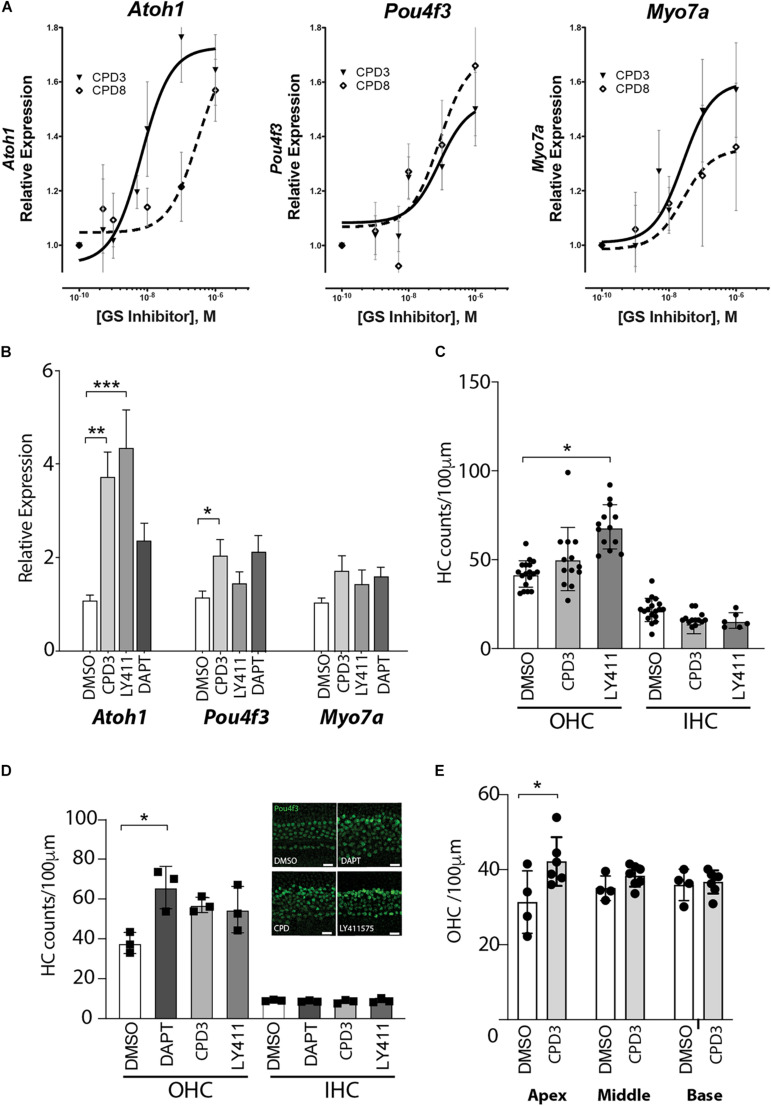
GSI-mediated induction of hair cell gene expression and *de novo* generation of hair cells in the neonatal cochlear sensory epithelium. **(A)** Gene expression changes of *Atoh1, Pou4f3*, and *Myo7a* in response to Notch pathway inhibition by CPD3 (*solid lines, triangles*) and CPD8 (*dashed lines, open square*) in otic spheres. Gene expression is reported as fold-change relative to carrier control-treated (DMSO, 0.05%). (*n* = 9–12). EC50 values are significantly different for Atoh1 (*p* < 0.05, *F*-Test). **(B)** Gene expression analysis for the hair cell genes *Atoh-1, Myo7a*, and *Pou4f3*. DMSO control *n* = 13, CPD3 *n* = 12, LY411575 *n* = 9, DAPT *n* = 6 from 2–4 independent experiments. OC explants were cultured for 4 days with/without GSI prior to lysis and analysis. One-way ANOVA with Dunnett’s multiple comparison test, each condition compared to DMSO control. (*p < 0.05, **p < 0.005, ***p < 0.001). **(C)** Cell counts of Atoh1 positive HCs (OHC left, IHC right) in the mouse cochlear sensory epithelium in the mid-apex region after 7 days in culture with different γ-secretase inhibitors. One-way ANOVA Dunnett’s multiple comparison test, each condition compared to DMSO control. (*p < 0.05; *n* = 3–5 independent experiments). **(D)** Quantification of Pou4f3 positive HCs (OHC left, IHC right) in the cochlear sensory epithelium from P2 rats in the apical domain after 8 days in culture with different γ-secretase inhibitors. One-way ANOVA with Dunnett’s multiple comparison test, each condition compared to DMSO control (*p < 0.05, *n* = 3). Representative immunohistological images of the apical domain after 8 days incubation (*inset*). scale bar = 20 μm. **(E)** Quantification of Pou4f3 positive OHCs in the apex, middle and basal domain of organ of Corti explants from rats after 4 days of CPD3 treatment compared to DMSO (*p < 0.05, unpaired *t*-test) *n* = 4–6 explants from 2 independent experiments.

CPD3 and CPD8 were further compared for their potency to induce hair cell gene expression in otic sphere assays (Oshima et al., 2009). Cells were first expanded in presence of growth factors, and in a second step differentiated by growth factor removal and GSI treatment. Both CPD3 and CPD8 significantly induced *Atoh1*, *Pou4f3*, and *Myo7a* expression in dose-responsive manner (*p* < 0.05; two-way ANOVA). However, CPD3 (IC50 = 6.7 nM) was more potent in *Atoh1* upregulation than CPD8 (IC50 = 302.8 nM; *p* < 0.05; two-way ANOVA). This indicated a modest advantage of CPD3 in eliciting expression of the early hair cell marker *Atoh1*, but similar potency on expression of some later markers of HC differentiation, *Pou4f3* and *Myo7a* (Figure 2A). From the results of two screening assays, CPD3 was selected for additional characterization studies based on its lower IC50 to induce *Atoh1/ATOH1* expression in the otic spheres and LS174T cells, respectively.

Subsequently, the analysis of gene expression changes induced by CPD3 was assessed in OC explants. As control GSIs, LY411575 and DAPT were used. CPD3 (1 μM) increased the expression levels of several hair cell genes: *Atoh1* (3.7 ± 0.54-fold; *p* < 0.005), *Pou4f3* (2.04 ± 0.35-fold; *p* < 0.05) and *Myo7a* (1.73 ± 0.33-fold; ns, one-way ANOVA with multiple comparison). Similar inductions were observed for LY411575 (5 μM): *Atoh1* (4.34 ± 0.82-fold; *p* < 0.001), *Pou4f3* (1.46 ± 0.26-fold, ns), and *Myo7a* (1.42 ± 0.31-fold; ns, one-way ANOVA with multiple comparison), as well as for DAPT (5 μM): *Atoh1* (2.36 ± 0.39-fold, ns) *Myo7a* (1.59 ± 0.21-fold, ns), and *Pou4f3* (2.14 ± 0.36-fold, ns) (Figure 2B).

In order to evaluate the potency of the compounds to induce *de novo* hair cell formation, OC explant cultures of the early postnatal organs were exposed to GSIs and hair cell numbers were quantified in cultures of mouse (Figure 2C) and rat tissue (Figures 2D,E). In both species, the GSIs increased OHC counts, whereas IHC counts remained unchanged. Here, OHC induction by CPD3 was moreover limited to the apical region (*p* < 0.05, unpaired *t*-test; Figure 2E).

**FIGURE 3:**
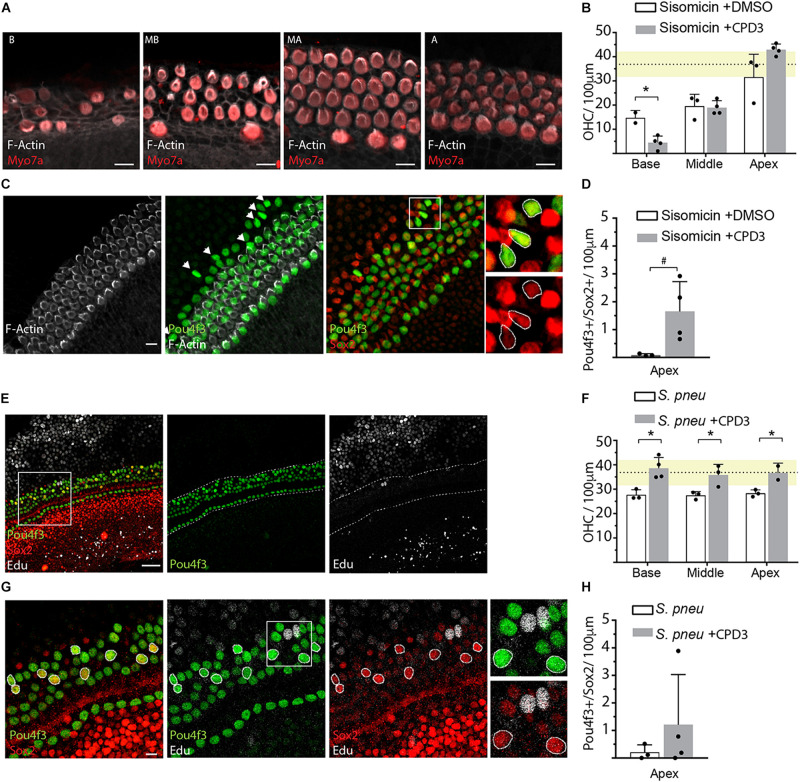
GSI treatment after ototoxic damage. **(A)** Representative immunohistological pictures of the base (B), midbase (MB), midapex (MA), and apex (A) of a sisomicin treated explant stained for hair cells (Myo7a), hair bundles (*F*-actin). Scale bars: 10 μm. **(B)** Quantification of OHCs in sisomicin-treated explants (2 h; 200 μM), 4 days after ototoxic insult, and cultured in presence or absence of CPD3. Dashed line shows the mean of the undamaged control explants with SD in yellow (*n* = 3–4 explants). **(C)** Immunohistological images of an apical OC turn after ototoxic damage with sisomicin and subsequent treatment with CPD3. Some cells co-expressed hair cell marker Pou4f3 and supporting cell marker Sox2 but did not show *F*-actin positive hair bundles (arrows). Scale bars = 10 μm. Boxed area is enlarged in the panels on the right showing merged and single channels. **(D)** Quantification of Pou4f3/Sox2 double-positive cells in the apex of sisomicin-exposed explants, treated with CPD3 or DMSO (ns, *n* = 3–4 explants). **(E)** Representative immunohistological images of the apical turn of the organ of Corti 4 days after exposure to *S. pneumoniae* and treatment with CPD3. Scale bar = 50 μm. **(F)** OHC quantification after bacteria-induced damage. Dashed line shows the mean of the undamaged control explants with SD in yellow. (*n* = 3,4 explants per condition). (*p* < 0.05, unpaired *t*-test, between CPD3 ± and each location). **(G)** Immunostaining of the organ of Corti apical domain for Pou4f3 and Sox2. Double-positive cells are highlighted with circles. EdU (*white*). Scale bar = 10 μm. **(H)** Quantification of Pou4f3/Sox2 double-positive cells in the apex after exposure to *S. pneumoniae*. (*n* = 3–4 explants per condition. ns, unpaired *t*-test with Welch’s correction). *p < 0.05.

To assess the regenerative potential of CPD3 in response to hair cell damage, treatment with CPD3 was tested in rat OC explants in two ototoxicity models: aminoglycoside exposure, specifically using sisomicin (Huth et al., 2015), and exposure to living *S. pneumoniae* (Perny et al., 2017; Erni et al., 2019). As reported previously, sisomicin caused a dramatic loss of hair cells, in particular OHCs, in the basal cochlear domain (Figure 3A) (Perny et al., 2017; Erni et al., 2019). Hair cell numbers in the apex remained comparable to control explants (dashed line/yellow shaded area), and increased OHC numbers elicited from CPD3 were not statistically significant, possibly due to this blunted baseline of damage (Figure 3B). Interestingly, an increase in Pou4f3+/Sox2+ double-positive cells at the apical side of the organ of Corti was detected, where 3rd Deiters’ and Hensen’s cells are located (Figure 3C). These double positive cells lacked hair bundles (*F*-actin) (Figure 3C, arrows), suggesting they were newly generated. Pou4f3/Sox2 double-positive cells were observed only in the apical domain of explants treated with CPD3 (Figure 3D) (*p* = 0.057, Unpaired *t*-test with Welch’s correction).

For bacteria-induced ototoxicity, the damage was moderate in these experiments (Figures 3E,F). Explants that were subsequently treated with CPD3 for 4 days showed more OHCs in all turns of the organ of Corti, compared to DMSO-treated organs, with a density similar to that of undamaged explants (Figure 3F). Again, some cells co-expressed Pou4f3 and Sox2, possibly indicating a process of direct transdifferentiation. This was supported by the lack of EdU incorporation (Figures 3E–G). Quantification of Pou4f3/Sox2 double-positive cells showed increased occurrence when explants were treated with CPD3 after damage, however, only in the apical domain. These effects did not reach statistical significance (Figure 3H).

### Hair Cell Formation in Whole Cochlea Culture

**FIGURE 4:**
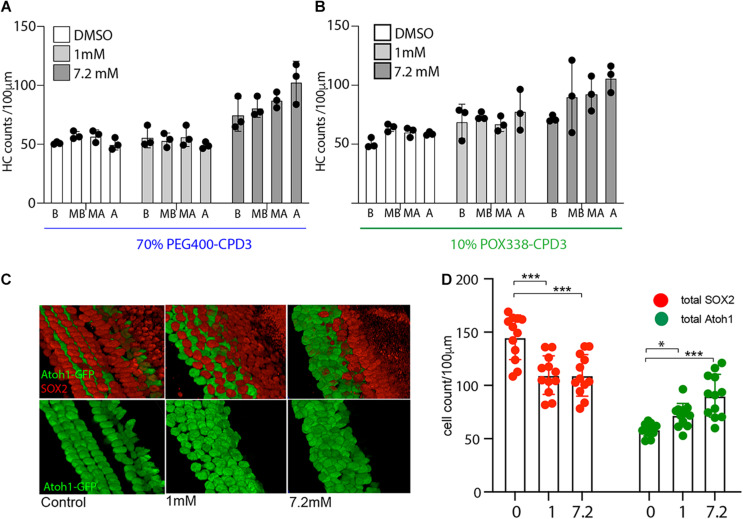
Hair cell quantification in whole cochlea culture. **(A)** Hair cell counts from whole cochea culture with directly applied CPD3 in 70% PEG400 (1 mM or 7.2 mM). *Atoh1*-GFP positive hair cells in different organ of Corti regions (*B*, base; *MB*, mid-base; *MA*, mid-apex; *A*, apex) (*n* = 3 independent experiments). **(B)** Hair cell counts from whole cochea culture with directly applied CPD3 in 10% POX338 (1 mM;, or 7.2 mM). *Atoh1*-GFP positive hair cells in different organ of Corti regions (*B*, base; *MB*, mid-base; *MA*, mid-apex; *A*, apex; *n* = 3 independent experiments). **(C)** Immunostained inner ear sensory epithelia following whole cochlea culture with direct application of CPD3 (1 and 7.2 mM; Vehicle, 10% POX338). 3D projections of confocal stacks at mid-base region of organ of Corti, Atoh1-GFP (hair cells, *green*) and Sox2 (supporting cells, *red*). **(D)** Quantification of *Atoh1*-GFP-positive (*green dots*) and Sox2-positive cells (*red dots*) after direct application of CPD3 (1 or 7.2 mM), or vehicle formulated in 10% POX338. (*p < 0.05; ***p < 0.001, one-way ANOVA with multiple comparison; *n* = 12; 3 independent experiments; all regions-base to apex).

To assess the permeability of the compounds across the round window membrane, a novel *ex vivo* model was devised to compare drug entry, after delivery to the exterior of the cochlear capsule, to resemble middle ear application. Specifically, CPD3 application was tested in organotypic culture of the entire otic capsule (Figure 4). CPD3, formulated at two different concentrations (1 and 7.2 mM) in 70% PEG400 or 10% POX338 (POX), was applied at the round window niche. Drug formulations were allowed to absorb briefly into the cochlea prior to the 48 h culture. Confocal microscope images of stained cells in four landmark regions (Base, Mid-Base, Mid-Apex, and Apex) revealed a dose-dependent increase in hair cell number (Atoh1-GFP positive cells) upon GSI treatment (Figures 4A–D). The total number of Atoh1+ hair cells, independent of the anatomical location, showed robust increases over control vehicles in both formulations. In the PEG400 formulation, CPD3 stimulated a 60.1% increase in Atoh1+ hair cells at 7.2 mM (87 ± 15 hair cells/100 μm) above vehicle-only (54 ± 5 hair cells/100 μm; *p* < 0.001, One-way ANOVA with Multiple comparison), but no change at the lower dose (1 mM; 54 ± 7 hair cells/100 μm). In the POX338 formulation, CPD3 stimulated Atoh1+ hair cell significant increases both in the low dose (23.3%) and at the high dose (Figure 4D, *green dots*; 54.4%; from 59 ± 6 hair cells/100 μm in vehicle-only, 72 ± 11 hair cells/100 μm at 1 mM and 90 ± 20 hair cells/100 μm at 7.2 mM; *p < 0.05; ***p < 0.001, one-way ANOVA with multiple comparison). For the POX338 formulation, we further characterized the number of Sox2+ supporting cells in the sensory epithelium. A significant decrease in supporting cell (Sox2+) counts was concomitant to the increase in Atoh1+ hair cells (Figures 4C,D, *red dots*; ***p < 0.001, one-way ANOVA with multiple comparison), in agreement with a previously reported transdifferentiation mechanism (Bramhall et al., 2014). Also in this model, we observed a trend for new hair cells to be greater toward the apex than the base in agreement with the results obtained with the OC organotypic culture and indicated GSI exposure was throughout the entire length of the organ of Corti length during the culture period.

### CDP3-Induced Hair Cell Differentiation in Cochlear Organoids

**FIGURE 5:**
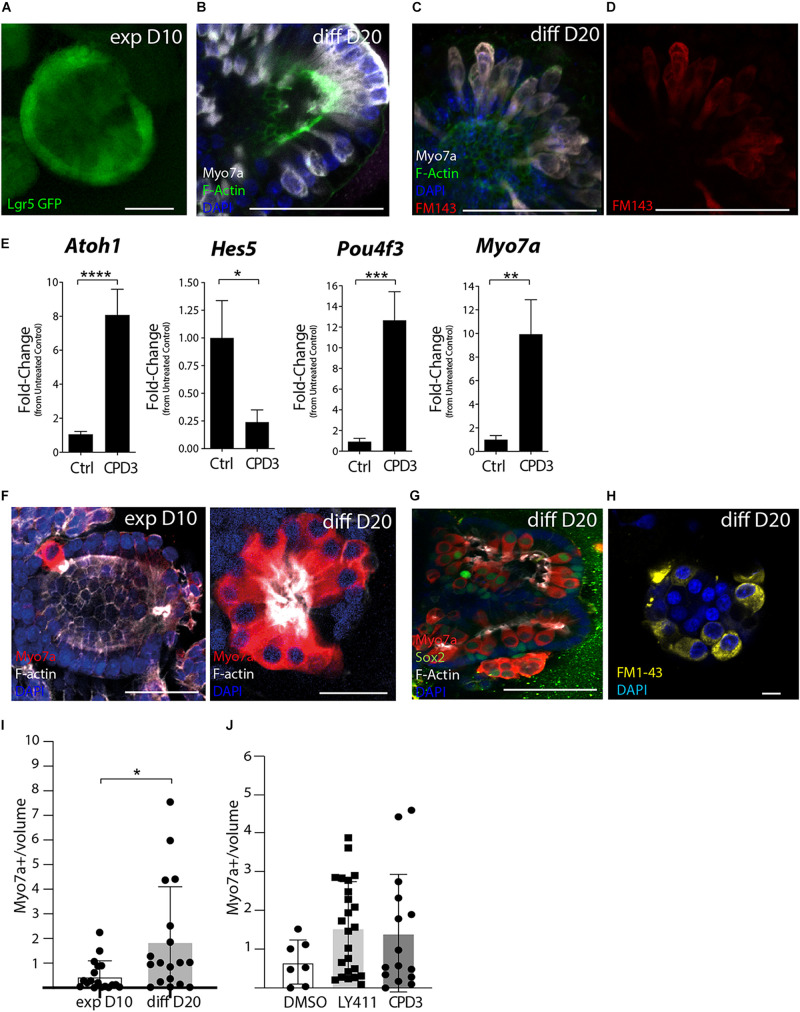
Assessment of GSI effects in cochlear organoid culture. **(A)** Representative example of Lgr5-GFP organoids after 10 days expansion (*exp D10*). Scale bar = 50 μm. **(B)** Immunostaining Lgr5-GFP organoids at 20 days of differentiation (*diff D20*) for Myo7a (*white*) and *F*-Actin (Phalloidin, *green*). Scale bar = 50 μm. **(C,D)** Incorporation of FM1-43-FX in organoid exposed to the dye for 30 s, subsequently fixed and immunostained for Myo7a (*white*) and *F*-Actin (*green*). Scale bar = 50 μm. **(E)** Gene expression analysis by RT-PCR of hair cell genes (*Atoh1, Pou4f3*, and *Myo7a*) and supporting cell gene (*Hes5*) in organoids derived from Atoh1-GFP mice at the end of differentiation. Ctrl (DMSO) and CPD3 (5 μM; *p < 0.05, **p < 0.005, ***P < 0.0005, ****p < 0.0001; Unpaired *t*-test; *n* = 14). **(F)** Representative example of organoids derived from the P2 rats cochlear sensory epithelium immunostained for the hair cell markers Myo7a (*red*) and *F*-Actin (*Phalloidin, white*) at day 10 of expansion (*exp D10*) and after day 10 of differentiation (*diff D20*). Scale bar = 25 μm. **(G)** Representative immunostaining at day 20 of differentiation for Myo7a, Sox2, and *F*-Actin. single *Z* stack. Scale bar = 50 μm. **(H)** FM1-43 uptake (*yellow*) at day 20 of differentiation. Scale bar = 10 μm. **(I)** Quantification of hair cell numbers *in vitro* (normalized for the volume of the organoids in mm^3^ (×10^–5^) at 10 days of expansion (*exp D10*) or at 20 days of differentiation in the presence of CPD3 (*diff D20*) *n* = 18 per group (*p* < 0.05; unpaired *t*-test with Welch’s correction). **(J)** Relative hair cells at the end of differentiation (normalized for organoid size). Total hair cells per organoid prior to normalization: DMSO (9.4 ± 11.2; *n* = 7), CPD3 (22.8 ± 18.0; *n* = 24), and LY411575 (17.17 ± 20.9; *n* = 15).

CPD3 was then tested in cochlear organoid culture (McLean et al., 2017; Lenz et al., 2019). In this model, supporting cells/progenitors are first expanded and subsequently differentiated to hair cells by growth factors withdrawal and addition of CHIR99021, to activate Wnt signaling, and inhibition of Notch, by GSI. Previously, LY411575 was used in similar experiments (McLean et al., 2017; Lenz et al., 2019). The use of Lgr5-GFP reporter mice enabled verification of Lgr5 induction during expansion (Figure 5A). Within 10 days of differentiation, cells of hair cell phenotypes were observed (Figure 5B), which also displayed uptake of the styryl dye FM1-43, an indirect readout for functional hair cells (Figures 5C,D).

Evaluating CPD3 efficacy to induce hair cell formation during the differentiation phase was assessed by gene expression changes of hair cell and supporting cell specific genes by RT-PCR. For this specific set of experiments, Atoh1-nGFP mice were used to facilitate the verification of hair cell induction prior to cell lysis for RNA isolation (data not shown). While hair cell genes (*Atoh1; Pou4f3*; and *Myo7a*) were upregulated, supporting cell genes (*Hes5* and *Lgr5*) were as expected downregulated. Sox2 mRNA expression was, however, not changed in this time-frame (Supplementary Figure 1). CPD3 (5 μM) induced Atoh1 expression (8.07 ± 5.6-fold; *p* < 0.0001, unpaired *t*-test), Pou4f3 expression (12.65 ± 11.07-fold; *p* < 0.0005, unpaired *t*-test) and Myo7a (9.9 ± 10.5-fold; *p* < 0.005, unpaired *t*-test) compared to untreated samples. Hes5 expression was downregulated fourfold (0.24% ± 0.4; *p* < 0.05 unpaired *t*-test) (Figure 5E). When compared to LY411575, comparable effects were observed for hair cells and supporting cell gene markers (Supplementary Figure 1).

Cochlear organoids derived from the rat sensory epithelium allowed corroboration of CPD3 to induce hair cell formation in another species model (Figures 5F–J and Supplementary Figure 2). As above, successful proliferation of Sox2+ progenitors (Supplementary Figure 2) was followed by cellular differentiation to generate Myo7a+ hair cells (Figures 5F–H). Hair cells demonstrated the ability to incorporate FM1-43, suggesting their functional differentiation in rat organoids also (Figure 5H). Interestingly, a subset of organoids contained cells double positive for Myo7a and Sox2, suggesting intermediate phenotypes during the transdifferentiation from supporting cells or immaturity (Figure 5G). The addition of CPD3 significantly increased hair cell numbers during differentiation (*p* < 0.05 Welch’s test) (Figure 5I), and CPD3 was then compared to LY411575 in the differentiation phase. For both compounds, higher numbers of hair cells per organoid could be differentiated relative to vehicle-treated samples, however, this did not reach statistical significance (Figure 5J). Some hair cells could also be identified when no GSI was added to the differentiation medium (DMSO group), with numbers comparable to what was obtained at the end of the expansion phase. To adjust for variation in size of the organoids, hair cell counts are expressed as relative to the organoid volume.

### Cochlear Organoids Hair Cells Are Sensitive to Sisomicin-Induced Damage

**FIGURE 6:**
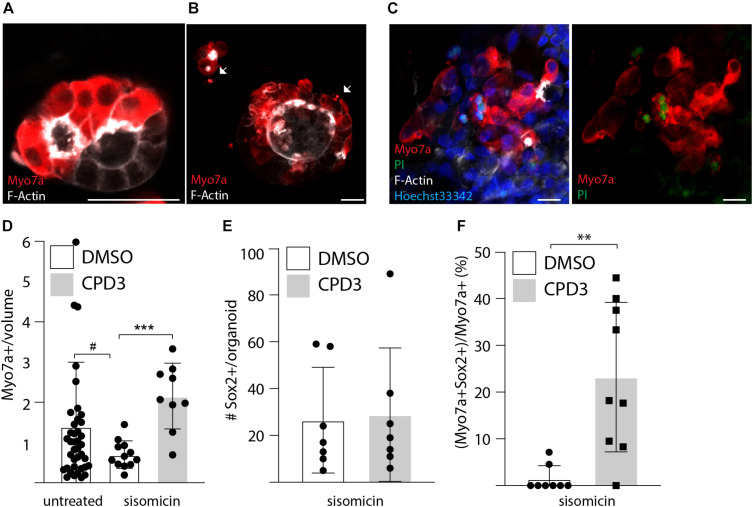
Ototoxic assay in cochlear organoid. **(A)** Immunostaining of untreated organoids for Myo7a and *F*-Actin at day 24. **(B)** Immunostaining of organoids for Myo7a and *F*-Actin at day 24 after exposure for 2 h to sisomicin (500 μM) on day 20. Cells presenting signs of apoptosis are labeled with arrows. **(C)** Propidium Iodide incorporation at day 24 is seen in a subset of hair cells after exposure to sisomicin. Scale bar: 25 μm. **(D)** Quantitative analysis of hair cells survival 1 week after sisomicin exposure [HC number relative to the organoid volume in mm^3^ (×10^–5^)]. Control: *n* = 40; sisomicin: *n* = 12; sisomicin + CPD3; *n* = 9 (^#^*p* = 0.052; ^∗∗∗^*p* < 0.001; unpaired *t*-test with Welch’s correction). **(E)** Quantification of Sox2 positive cells after sisomicin damage and CPD3 exposure (*n* = 7). **(F)** CPD3 effects on the percentage of Myo7a positive cells co-expressing Sox2 (^∗∗^*p* < 0.001, unpaired *t*-test with Welch’s correction; *n* = 8–9).

Finally, the regenerative potential of CPD3 was assessed with rat cochlear organoids testing the efficacy of CPD3 to transdifferentiate any remaining supporting cells after ototoxic damage. Sisomicin (500 μM) was applied for 2 h at day 20 of differentiation, followed by 4–7 days culture in absence or in presence of GSIs. Qualitative assessment of the ototoxic effect of the aminoglycoside showed features of damage, including abnormal morphologies, cell shrinkage and uptake of propidium iodide in Myo7a positive cells, consistent with sisomicin-induced cell death (Figures 6A–C). A decrease in Myo7a+ hair cells was detected 7 days following sisomicin damage (Figure 6D). From the baseline of sisomicin-treated hair cell numbers, a significant increase in Myo7a+ hair cells was restored in organoids that were cultured in presence of CPD3 (*p* < 0.001, unpaired *t*-test with Welch’s correction) (Figure 6D). While the total number of Sox2 positive cells in the organoid did not change in response to CPD3 (Figure 6E), the number of Myo7a+/Sox2+ hair cells did increase (*p* < 0.001, unpaired *t*-test with Welch’s correction) (Figure 6F). Together these data indicated that, GSI treatment did induce *de novo* hair cell formation from supporting cells in the sisomicin-damage organoid model.

## Discussion

Reactivation of signaling pathways that contribute to hair cell development during embryonic and fetal stages is one of the approaches under investigation to trigger tissue regeneration of the inner ear sensory epithelia. This strategy targets supporting cells, given the common developmental origin, and aims to force their *de novo* differentiation to hair cells (Roccio et al., 2019). With this rationale, Notch signaling was manipulated in a set of experiments to characterized the *in vitro* responses of the cochlear sensory epithelium to chemical Notch inhibition.

The activities of two novel GSIs were assessed in this study and compared to the commercially available inhibitors. The new compounds displayed intermediate potency to previously reported GSIs in terms of IC50 for *ATOH1* induction. Still they induced comparable levels of hair cell generation *ex vivo* in all the tested models. *De novo* hair cell induction appeared to be driven by the transdifferentiation of supporting cells. Using *ex vivo* culture and pharmacokinetic studies, it was possible to evaluate their permeability through the round window membrane, assess different formulations, and determine the local concentration of these compounds into the inner ear. Ultimately, CPD3 was further selected as the new chemical entity for a phase1/2 clinical trial for mild-moderate SNHL (REGAIN trial^1^).

Gamma secretases have additional cellular targets other than Notch receptors, including the amyloid precursor protein (APP), for which they were initially designed, ERBB4, *E*-cadherin and others (Andersson and Lendahl, 2014). Specifically for the inner ear, the GSI effects observed in early postnatal stages are comparable to genetic Notch suppression (Ni et al., 2016a,b). In a direct comparison between GSI treatment (DAPT) and Notch blocking antibody *ex vivo*, similar levels of hair cell gene expression and *de novo* hair cell induction were reported (Maass et al., 2015). GSIs such as LY411575 have greater potency than the older transition state analogs and DAPT-type peptidomimetics (Han and Shen, 2012). However, the increased potency comes with some off-target effects, such as non-selectivity toward proteases and can result in inhibition of signal peptidases (Weihofen et al., 2003) or the proteasome (Han and Shen, 2012). The choice of the novel molecules assessed in this study was primarily based on the ability to inhibit Notch signaling and reduce potential off target effects. The intermediate potency of CPD3 and CPD8 and their concentration in perilymph, notably higher than the IC50 for *ATOH1* induction, were therefore positively evaluated. Finally, local delivery to the inner ear has the advantage to likely minimize systemic side effects (Andersson and Lendahl, 2014).

The initial pharmacology of the different GSIs was characterized in a human cell line that responds to Notch inhibition and allows for quantitative comparisons of IC50 for the upregulation of *ATOH1*. The phenotypic assays that followed were performed in inner ear tissues intended to increasingly represent targets of therapeutic treatment. In each of the assays, the concentration ranges used were based on the concentrations achieved in pharmacokinetic tests (Figure 1). Using organotypic cultures, CPD3 upregulated the expression of hair cell genes in the tissue and resulted in an increased number of hair cells, in particular OHCs in the apical region of the sensory epithelium, in agreement with previously reported GSI studies (Korrapati et al., 2013; Li et al., 2015; Maass et al., 2015; Ni et al., 2016b). CPD3 increased hair cells in undamaged cultures and also after ototoxic damage induced by sisomicin. In the case of *S. pneumoniae* exposure, we observed higher numbers of putative novel hair cells, co-expressing *Pou4f3* and *Sox2* in the apical domain. However, hair cell counts were increased in all cochlear regions after GSI treatment. It remains unclear which mechanism may contribute to this increase and whether GSIs may exert some partial otoprotective function in this model.

While the reason for the higher regenerative potential of the apical domain of the cochlear sensory epithelium in early postnatal ears is likely related to the younger age of these cells relative to the hair cells at the base (Driver and Kelley, 2020), these differences in plasticity are unlikely to hold for the adult. This regional effect may pose a problem for hair cell regeneration as OHCs in the base are most vulnerable to damage (Liberman, 1990; Sha et al., 2001; Perny et al., 2016; Perny et al., 2017) resulting in predominant high-frequency hearing loss.

The *in vitro* findings with rat explant cultures supported a model where hair cells are generated by transdifferentiation of supporting cells as immature hair cells expressing both Pou4f3 and Sox2, lacking hair bundles were identified, but these cells did not incorporate EdU (Figure 3). This is in contrast with mitogenic responses observed with murine tissue by us (Roccio et al., unpublished data) and others (Li et al., 2015; Ni et al., 2016b). Further, different supporting cell types responded to GSI treatment and damage. In explants exposed to sisomicin, newly-formed hair cells were detected adjacent to the 3rd row of hair cells and therefore likely originated from transdifferentiation of the 3rd Deiters’ cells/Hensen’s cells. In explants exposed to live *S. pneumoniae*, newly-formed hair cells were located at different locations across the medial-lateral axis of the sensory epithelium. It is therefore plausible that different ototoxic damage models may activate different supporting cell types, modifying their response to regenerative molecules. Interestingly, ectopic hair cells were not observed in the greater epithelial ridge, nor in the inner hair cell region after GSI exposure.

Transdifferentiation of supporting cells to hair cells without replenishment of the supporting cell pool may alter the epithelial organization and therefore *per se*, impact the organ functionality. Different studies have focused on simultaneous induction of proliferation and differentiation of supporting cells to mimic a regenerative response (Ni et al., 2016a,b; Shu et al., 2019). Restoration of the original cellular organization remains a major challenge to be solved in order to enable proper restoration of organ function.

Organotypic cultures of the postnatal organ of Corti are well-established and widely used tools to evaluate *in vitro* responses to drugs as they can be directly exposed to selected concentrations of compounds under controlled conditions. They also enable the assessment of the anatomical location and the type of cells that are affected by the treatment. However, any approach for *in vivo* delivery will likely not match the high and precise doses that can be tested *in vitro*. In order to complement the pharmacological data, a different *ex vivo* model of the whole cochlea from mice was established. The model aimed to demonstrate that CPD3 could induce an increase in hair cell number by local delivery and to corroborate the preclinical pharmacokinetic tests documenting drug absorption into the perilymph. CPD3 was tested in two vehicle formulations: 70% PEG (Mizutari et al., 2013) and 10% POX (Gausterer et al., 2020), designed to increase the residence time *in vivo*. In both cases, the highest concentration of the compound led to a basal-to-apical gradient of increased hair cell number. A decrease of Sox2+ cells reaffirmed that GSI entered the cochlea and induced hair cell formation, predominantly through transdifferentiation. The data reported here are obtained from P1–2 mice. While the model provides an initial, complementary approach to test drug permeability and efficacy, it remains highly challenging to establish similar *ex vivo* culture systems from older animals.

Finally, cochlear organoid models (McLean et al., 2017) were successfully employed to compare the effectiveness of GSI in a range of experiments testing hair cell regeneration. The culture methods rely on a primary phase of cochlear progenitor expansion followed by differentiation to hair cells by concomitant Wnt activation and Notch inhibition (McLean et al., 2017; Roccio et al., 2018; Lenz et al., 2019). This enabled a side-by-side comparison of GSIs. In this case, the efficacy of hair cell induction of CPD3 was comparable to LY411575, both in terms of upregulation of hair cell genes, as well as the number of derived hair cells at the end of the differentiation period. Comparable findings were obtained from mouse and rat cultures. While a direct comparison was not conducted, rat cochlear organoids seemed to have a higher proportion of Sox2+ cells at the end of differentiation. Hence, this provided a convenient model to test supporting cell-dependent hair cell regeneration. The sensitivity to sisomicin in rat organoids suggested a functional cochlear hair cell phenotype. Interestingly, hair cells lost from the ototoxic insult were replenished after CPD3 treatment (Figure 6).

Organoid culture models have the drawback that they lack information concerning the precise cell type and anatomical location where damage and regeneration occur, as supporting cells derived from the entire cochlear duct are isolated, pooled and cultured in homogeneous conditions. Furthermore, the media composition has been selected to maximize the proliferative and stem-like features of these cells, and findings obtained *in vitro* may not be representative of *in vivo* responses obtained in intact organs with single drugs. It is, however, possible to maintain these cultures for a prolonged time. Here, we tested up to 1 month (20 days of differentiation 7 days after damage). Optimization of the conditions may enable further maturation to late postnatal stages. In contrast, *ex vivo* cultures of the OC tend to lose tissue organization once cultured for a prolonged time, and late postnatal tissue cannot be directly isolated undamaged from the ossified inner ear. Benchmarking *in vitro* derived hair cells and supporting cells from the organoid cultures against primary tissue from late postnatal and adult organs will be pivotal to better understand the advantages and limitations of this model (Burns et al., 2015; Ranum et al., 2019; Yu et al., 2019; Hoa et al., 2020).

Furthermore, validation of these strategies on human tissue may further provide robust evidence for their clinical translation. While human fetal and adult (postmortem or surgically derived material) can be used for important validation studies, their limited accessibility in fact strongly hampers the use in routine or high throughput experimental settings (Chen et al., 2007; Roccio et al., 2018; Taylor et al., 2018). Generation of human cell types using stem cell technologies may provide alternative and scalable tools for *in vitro* testing of drug therapies (Koehler et al., 2017; Roccio, 2020). In conclusion, while the tested GSI reliably enhanced *de novo* hair cell formation *in vitro* in all the presented assays, a major future hurdle will be to generate advanced models that are representative of mature or aged tissues to study disease and regeneration in these settings prior to the translation to more complex *in vivo* models.

## Data Availability Statement

The original contributions generated for this study are included in the article/Supplementary Material, further inquiries can be directed to the corresponding authors.

## Ethics Statement

The animal study was reviewed and approved by the Animal Care and Experimentation Committee of the Canton of Bern, Switzerland [license BE 142/16 (SL); BE119/12 (MR)] and followed the Swiss national guidelines for the performance of animal experiments, or under an approved institutional protocol according to National Institutes of Health guidelines, or under animal protocols approved by the Animal Studies Committee of Languedoc Roussillon that comply with French legislation and European Directives.

## Author Contributions

SE, CP, GF, MB, JG, KL, and MR planned and executed experiments and collected and analyzed data. SE and JG performed statistical analysis and contributed significantly to manuscript writing. DG, SL, AE, and MR study design, project supervision, funding, and manuscript writing. All authors contributed to the article and approved the submitted version.

## Conflict of Interest

JG and KL are employed by Audion Therapeutics. AE is a founder and consultant to the company. Part of the study at the University of Bern was sponsored by Audion Therapeutics. The remaining authors declare that the research was conducted in the absence of any commercial or financial relationships that could be construed as a potential conflict of interest.

## Publisher’s Note

All claims expressed in this article are solely those of the authors and do not necessarily represent those of their affiliated organizations, or those of the publisher, the editors and the reviewers. Any product that may be evaluated in this article, or claim that may be made by its manufacturer, is not guaranteed or endorsed by the publisher.

## Footnotes

1
https://www.regainyourhearing.eu/
