# Supplementary material for: Hair Cell Generation in Cochlear Culture Models Mediated by Novel γ-Secretase Inhibitors
Source: Front Cell Dev Biol. 2021 Aug 13;9:710159. doi: 10.3389/fcell.2021.710159 (PMC8414802; doi:10.3389/fcell.2021.710159)
Supplement: Supplementary file 1 [file Data_Sheet_1.PDF]

Supplementary figure S1 Erni et al

Supplementary fig S1 Erni et al

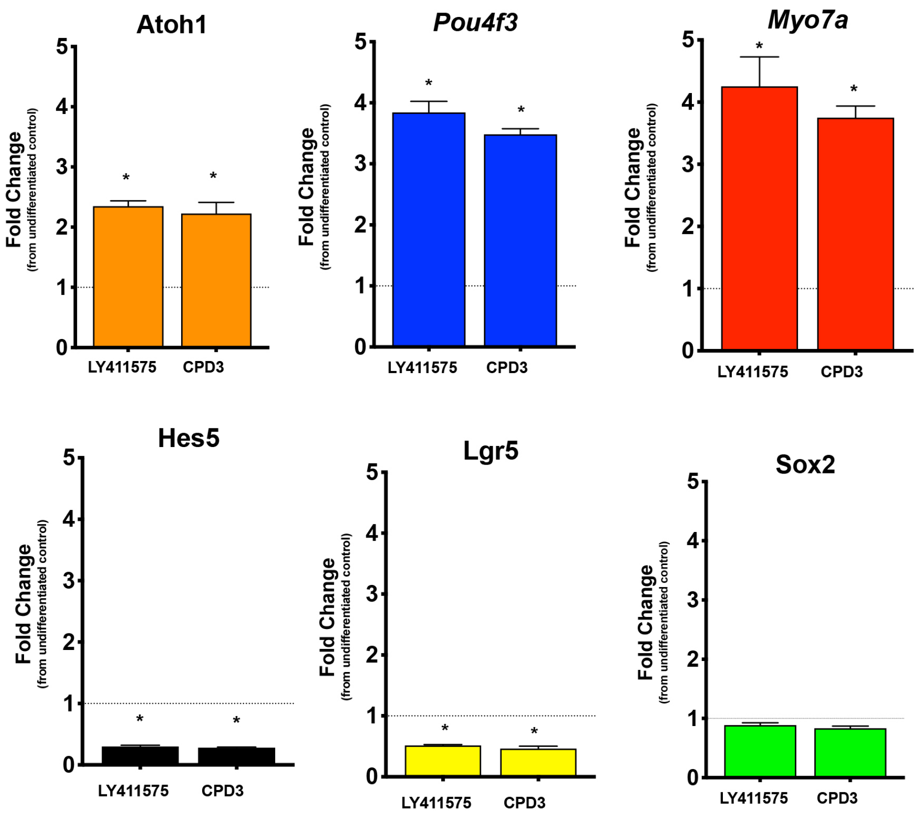

Fig S1.

Gene expression analysis by RT-PCR of hair cell genes (*Atoh1, Pou4f3, Myo7a*) and supporting cell markers (*Hes5, Lgr5 and Sox2*) in organoid culture at the end of differentiation. Fold changes to control (NO-CHIR/NO-GSI) are shown. Comparison between LY411 (400nM) and CPD3 (400nM), -treated sample is shown, both treated concomitantly with CHIR. N=3 independent experiments.

Supplemenrary Figure S2 Erni et al

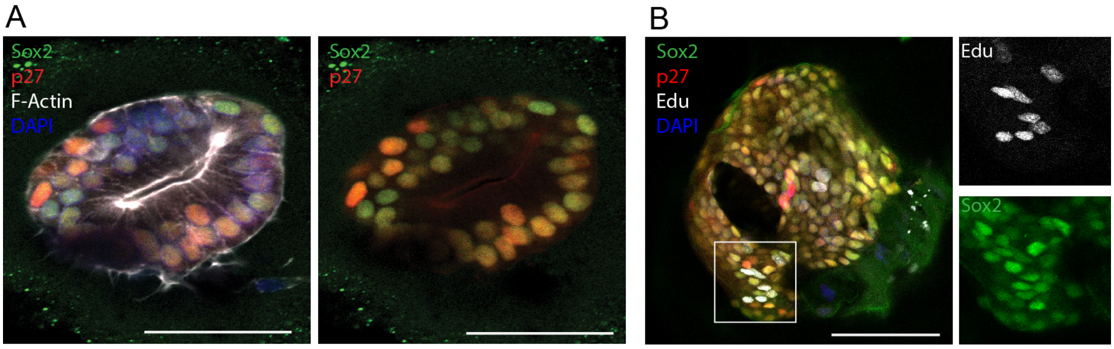

Supplementary figure S2

A. Representative example of cochlear organoids from the rat cochlear sensory epithelium at day 10 of expansion immunostained for the supporting cell markers p27/Kip1 and Sox2 and F-actin labeling. Scale bar = 50 μm.

B. Incorporation of Edu during the expansion phase in Sox2+ cells. Scale bar = 100 μm
